# Essential Requirements and Relevant Technologies for Load-Bearing 3D-Printed Transtibial Prosthetic Sockets and Their Components: State-of-the-Art Review

**DOI:** 10.2196/73065

**Published:** 2025-12-17

**Authors:** Erika Dagge, Breda Clancy, Gavin Keane, Brian Casey, Declan Devine

**Affiliations:** 1 Department of Mechnical and Polymer Engineering, Technological University of the Shannon: Midlands Midwest, University Road, Athlone, Co. Westmeath, N37 HD68, Ireland, 090 6468291; 2 CÚRAM Research Ireland Centre for Medical Devices, Biomedical Sciences, University of Galway, Galway, Ireland; 3 Atlantic Prosthetic Orthotic Services Ltd, Galway, Ireland; 4 Department of Humanities, South East Technological University, Carlow, Ireland

**Keywords:** load-bearing, prosthesis, transtibial, additive manufacturing, digitalization, 3D printing, prosthetic socket

## Abstract

**Background:**

The manufacture of load-bearing prosthetic lower limb sockets is traditionally reliant on skilled technicians working with qualified clinicians to create bespoke solutions. While this approach is effective and, in some situations, necessary, the appeal of a sustainable, efficient, and digitalized production solution cannot be ignored. The focus of additive manufacturing (AM) is typically on low-weight-bearing prostheses, which can be misleading for clinics attempting to adopt AM solutions for clientele with weight-bearing or activity-level needs.

**Objective:**

This review aims to offer readers a way to approach AM for load-bearing requirements as opposed to non–load-bearing counterparts. The use cases of AM for the production of load-bearing transtibial prosthetic sockets and components are reviewed to highlight current trends, protocols, and standings.

**Methods:**

By reviewing publications from the past 25 years, this state-of-the-art review highlights the key requirements and technologies relevant for load-bearing transtibial prosthetic sockets specifically.

**Results:**

The most commonly used AM solutions for commercial use, such as selective laser sintering and binder jetting through Multi Jet Fusion, are outlined. As these solutions are most often paired with the structural testing standard International Organization for Standardization 10328, their relevance for evaluating the strength and durability of lower limb sockets is also discussed. Clinician and technician experiences of digitalized ways of working within the prosthetic industry for load-bearing applications are outlined.

**Conclusions:**

Observations of adoption barriers of AM solutions are brought to light, focusing on clinician and technician education, skill set, exposure to innovative technologies, and trust in the regulation of digital processes in a clinical and technical environment.

## Introduction

### Background

**Figure 1.**
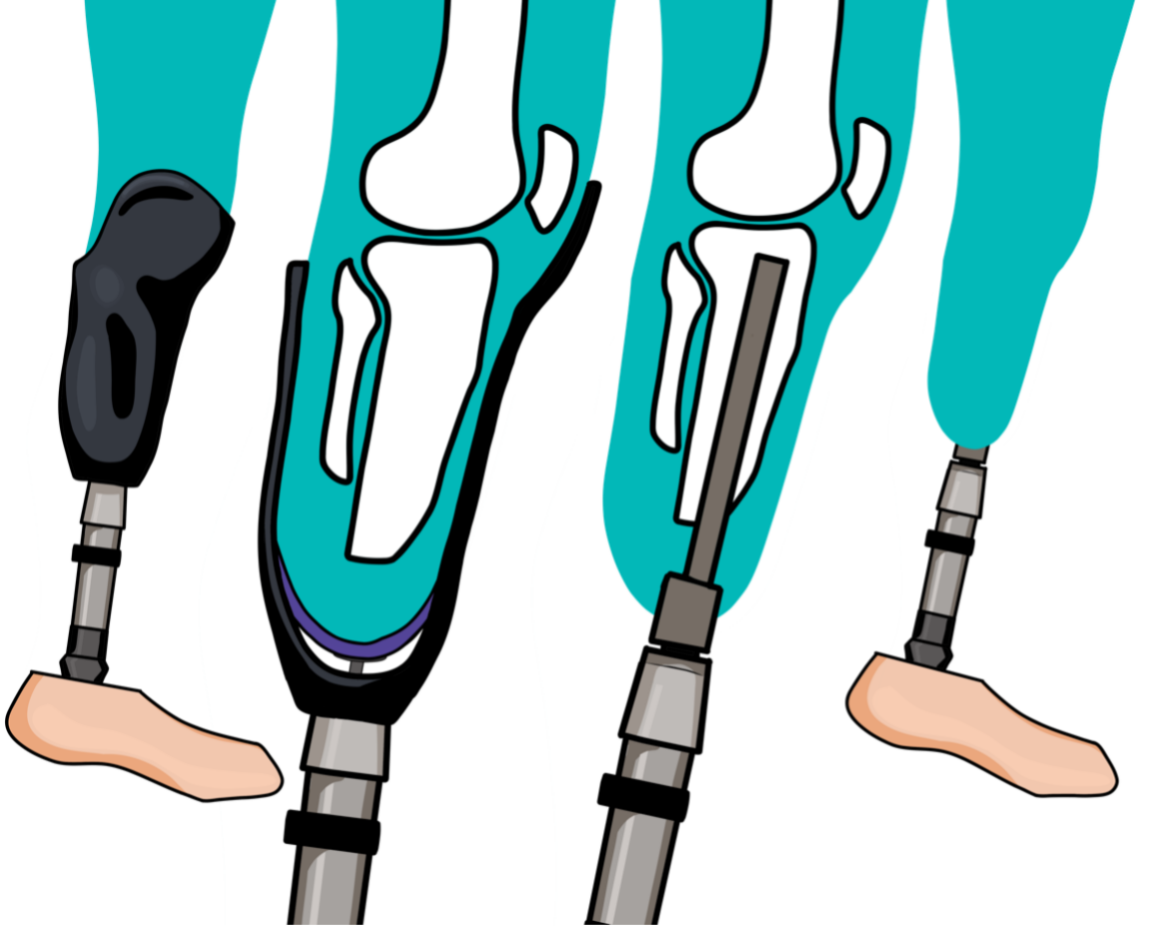
Transtibial prosthetic devices, observing an external approach using a socket component (left) and an internal osseointegrated approach (right).

A prosthesis, or prosthetic device, acts as a replacement for a lost or missing limb or body part, with functionality as a key added value [1]. The function of a prosthesis can range from basic motion to combined rotation and hinging. The device may contain technological parts, which can rotate, stabilize, and compress. These parts can be manipulated through the interpretation of electronic pulses in the body recorded from microprocessors [2,3]. With many components making up the assembly of a prosthesis, there must be a connection point between the existing limb and the artificial device to provide functionality. In the case of a lower limb prosthesis, this connection can be made through the implementation of an osseointegrated implant or using an external component known as a socket [4]. Depending on the degree of limb loss, the attachment of a prosthesis can be made above the knee (transfemoral) or below the knee (transtibial). The focus of this review is relevant to the implementation of transtibial external prosthetic sockets specifically. Figure 1 provides a visual comparison of an external transtibial prosthesis using a socket component and an implanted osseointegrated transtibial prosthesis.

When manufacturing transtibial sockets, a traditional, skill-based workflow has determined shape, weight, function, and fit for the end user [5]. The procedure involves a hands-on approach, convenient for control and accuracy for the clinician and technician. However, this approach has been proven to be costly, wasteful, and time-consuming for both material and clinical time usage [6]. For these reasons, the manufacturing workflows have been evolving with digitalization and technological advances [7].

The exploration of additive manufacturing (AM) processes is currently showing success for non–load-bearing prosthetic devices. There are a variety of solutions available for the AM of upper limb prostheses, each using a variety of materials [8]. With the demand present in low-income countries, there have been volunteer assistive device designers actively contributing to the AM of prosthetic devices in the past few years [9].

For load-bearing devices, however, validating the efficacy of 3D-printed devices remains a challenge, compounded by the customized nature of each prosthesis. A variety of AM processes have been used to create load-bearing transtibial prosthetic sockets, with some methods bearing more strength and durability than others [10,11]. In Kim et al’s [12] review of 1023 studies on this topic, the considerable amount of heterogeneity between studies (in terms of materials and alignment used) meant that the absolute values for failure could not be established for AM. As such, these values also could not be compared to traditionally made devices. Kim et al [12] highlight that the reviewed studies provide limited evidence for AM used in a clinical setting, and those that do so have only evaluated the durability of these prostheses for a period of 2-6 weeks [12].

As digitalization becomes more prevalent, positive effects, such as time efficiency, improved patient care, and sustainability have been noted [13]. Positive convergence between prosthetics and orthotics (P&O) and a variety of industries can develop and progress new and innovative technological solutions. This has the potential to evolve our society from a global cultural perspective and improve the quality of life for the end user [14]. However, with traditional methodologies still being at the forefront of education and day-to-day workflows, it is difficult for clinicians and technicians to value the benefits of 3D printing in their roles [15].

### Traditional Manufacturing of Transtibial Prosthetic Sockets

There are a variety of transtibial socket types varying for each patient, dependent on their weight class, bone structure or definition, and limb condition (scarring, skin vulnerable to breakdown, etc) [5]. Many transtibial sockets created are patellar tendon bearing. There are a variety of suspension and load-bearing conditions branching from this socket type to give the most comfortable and functional solutions for each patient.

**Figure 2.**
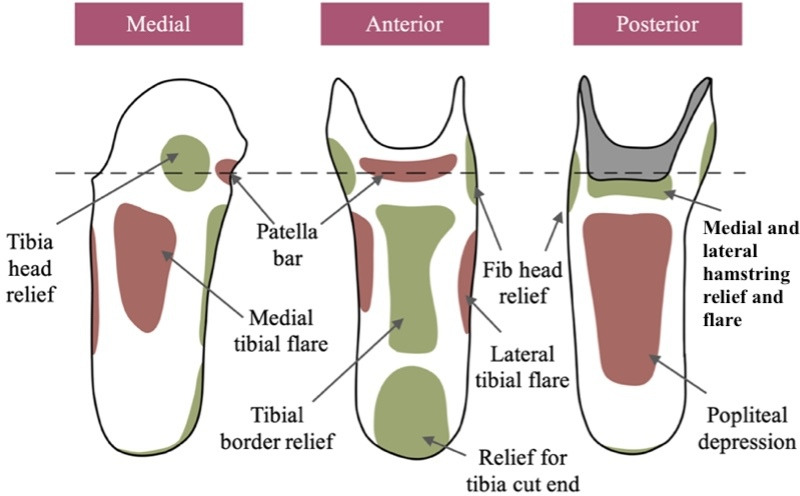
Relevant landmarks for transtibial socket rectification, where green areas are typically areas of pressure alleviation. Fib: fibula.

In traditional manufacturing of clinical prostheses, it is the duty of a qualified clinician to carry out socket rectification (manipulation of the socket form to optimize distributed loading onto the limb) [16,17]. Traditionally, this rectification process is performed by hand on a plaster replica of the patient’s residual limb by the clinician. When being worn, the socket is considered a total-contact device. However, loading should be avoided in areas, such as the fibula head, cut or distal ends of the tibia and fibula, and the anterior border of the tibia (shin) [18]. The rectification process involves shaping the socket form to alleviate pressure and load-bearing from these sites. The rectification process subsequently applies load-bearing areas to other appropriate and learned landmarks [5]. Figure 2 illustrates the basic landmarks and areas of a socket that may be altered during the rectification of a transtibial socket.

The material choice for traditionally made sockets for lower limb prostheses has evolved through the ages. Transitions from wooden and metal componentry to lightweight thermoplastics and reinforced composite polymers have occurred for sockets [19]. The use of carbon fiber as an incorporated material for sockets has enhanced device strength while maintaining its lightweight property. Each traditionally made socket can consist of varied materials, such as standard polyester and acrylic resin, laminates, and more complex composites with fabric inclusions [7].

With an understanding of the load-bearing characteristics of a transtibial prosthetic sockets in place, the following information aims to guide the reader into the process of digital processing and AM. The upcoming sections focus on relevant load-bearing testing, materials, and manufacturing requirements of load-bearing sockets that are applicable to traditionally made and AM sockets alike.

### Clinical Applicability of AM for Prosthetic Sockets

AM is the process of creating 3D physical objects using machinery hardware and computer-aided design data from modeling software [20]. Also referred to by its original title “3D printing” in many instances, all these processes use a layer-by-layer method of fabrication in order to construct a physical 3D form [20].

Although AM technologies have been used effectively in cosmetics and small orthotic devices, industry adaptation for load-bearing componentry has been slow to occur. This raises questions as to why AM is not being used in more P&O clinics worldwide. Within P&O, there is not only an added opportunity to personalize the original devices when using AM but also to decrease material wastage, clinical, and labor time. AM can also improve data collection and production rates [21]. Slow industry adaptation can be seen with new technologies in the P&O industry due to trust and establishment requirements, ethical concerns, and educational barriers to the usage of such devices or workflows [22]. An example of slow adaptation in this industry can be seen through the introduction of microprocessor knees, which, although developed in the 1980s, were not commercially viable until 1999 [23].

Nevertheless, the solution of AM has been recorded as viable and advantageous for the production of transtibial load-bearing sockets, showing considerable benefits when combined with a digitalized workflow in industry.

To create a transtibial socket using AM, the process typically entails a new digitalized process of manufacturing. In this process, digital technologies such as 3D scanning and online or computerized rectification and fabrication systems take over the physical plaster mold form capture [24].

**Figure 3.**
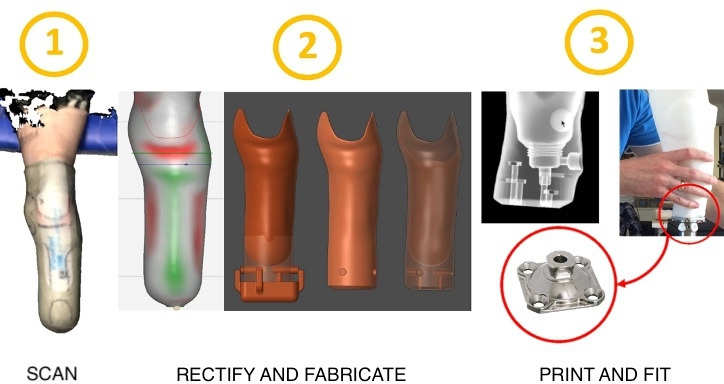
A typical digitalized process for 3D printing prosthetic sockets.

Figure 3 provides an overview of the typical steps involved in the digitalized process.

The need for physical storage space is significantly reduced as plaster casts become digitalized files. These files can be stored in secure computer storage systems, accessible to recognized employees of the company for 3D printing. However, without appropriate regulation, online data storage inevitably brings data protection issues and potential risks of personal data sharing to light [25].

A prosthetic team of clinicians, technicians, and administrative staff typically works together to decide on the appropriate AM solution and polymeric material, be it composite or noncomposite materials used. This typical production team is established for devices produced in-house or through external AM facilities. The implementation of this way of working has been found to be successful in improving business processes for prosthetic service providers and manufacturers [26-28].

In summary, with plaster limb casting translating to 3D scanning, companies have the opportunity to reduce the requirement of physical material and storage space for limb form capture. Digital rectification, used in place of traditional rectification, again offers the possibility to eliminate the use of plaster, as well as reduce the requirements of technician resources. Resin casting and thermoform fabrication, translating to AM technologies, lead to significantly fewer environmental and health hazards in the workplace if printed out-of-house. All of these innovative technologies have positive effects on the prosthetic manufacturing process, compared to their traditional counterparts [27].

### Regulatory Challenges of Lower Limb Prostheses

In the realms of regulation, a lower limb prosthesis is identified as a custom-made device and does not require a Conformité Européenne (CE) marking as an assembly. As with all medical devices, the regulations and protocols surrounding the design, manufacture, and rollout of prosthetic devices are strictly adherent to various key regulations. Table 1 is a summary of the key regulations for load-bearing prosthetic lower limb sockets [29-33].

**Table 1.** Regulations and standards relevant to load-bearing lower limb prosthetic sockets.

Standard/Regulation	Relevance	Requirements (nonexhaustive)
European Union Medical Device Regulations (EU MDR) 2017/745.	Identifies prosthetic devices as custom-made devices. Outlines the rules and requirements of manufacturers and the device itself.	Manufacturer’s statement. General safety and performance requirements (GSPR) adherence. Postmarket surveillance system. Implementation of appropriate quality management and risk assessment systems.
International Organization for Standardization (ISO) 13485:2016 - Medical devices - Quality management systems - Requirements for regulatory purposes.	Outlines a quality management system for the manufacturing and distribution of prosthetic devices.	Documentation of design, development, production, installation, and servicing of devices. Risk management, compliance, and documentation for devices. Traceability and documentation of materials, processes, and testing of devices.
ISO 22523:2006 - External limb prostheses and external orthoses (requirements and test methods).	Outlines testing requirements for external prosthetic devices, referencing strength, and material considerations.	Definition of terminology, performance, and safety requirements for devices. Biocompatibility of materials for devices. Labeling and device information requirements. Minimum structural and functional safety requirements of devices.
ISO 10328: Structural testing of lower limb prostheses.	Outlines the mechanical testing requirements of assembled prosthetic devices for CE^a^ marking and evaluation.	Unique test rig aligned to toe-off and heel strike phases of gait. Specified loading according to patient weight and activity level. Number of tests and cycles required for proof, ultimate and fatigue, and torsion testing. Alignment requirements for various assemblies and component inclusions.

a CE: Conformité Européenne.

It is the duty of the prosthetist or physician to determine the activity level (also known as “K level”) of a patient. This is determined in the consultation and assessment periods prior to allocating the patient a prosthesis [34]. A patient’s activity level can change over time through rehabilitation if deemed suitable by the prosthetist, during recurrent monitoring of the patient’s rehabilitation process [35]. Table 2 shows each K level with a descriptor of the ability of each patient [35].

The level of loading applied to a lower limb prosthesis is determined by the variety and quantity of CE-marked components used, as well as the level of design complexity (for CE-marked components only). ISO (International Organization for Standardization) 10328 determines the weight limit of the prosthesis wearer for the device being tested [36]. This weight limit is calculated through the testing of mechanical load capacities to ensure the safety and durability of all devices with varying patient activity levels [36]. Transtibial prosthesis assembly will have different loading requirements than that of a transfemoral device with knee joint componentry, although the alignment requirements for testing remain consistent for both. By maintaining consistent limitations and alignment, device componentry can be accurately compared for its changes in material and form. It is not stated how the individual socket and residual limb alignments may affect the consistency of testing results.

The load capacities of a lower limb prosthesis are addressed as “P Levels” within the standard ISO 10328. Ranging from P3 to P8, each level determines a separate weight limit dependent on the loading that each prosthesis can withstand. The standard also notes how factors such as lifestyle and body mass must be taken into account when determining which P level is reflective of the prosthesis use case. Table 3 offers the reader a breakdown of weight limits for each P level [36].

**Table 2.** K levels indicating the rehabilitation potential of amputees.

K Level	Descriptions
Level 0	Does not have the ability or potential to ambulate or transfer safely with or without assistance, and a prosthesis does not enhance their quality of life or mobility
Level 1	Has the ability or potential to use a prosthesis for transfers or ambulation on level surfaces at a fixed cadence. Typical of the limited and unlimited household ambulator.
Level 2	Has the ability or potential for ambulation with the ability to traverse low-level environmental barriers, such as curbs, stairs, or uneven surfaces. Typical of the limited community ambulator.
Level 3	Has the ability or potential for ambulation with variable cadence. Typical of the community ambulator who has the ability to traverse most environmental barriers and may have vocational, therapeutic, or exercise activity that demands prosthetic usage beyond simple locomotion.
Level 4	Has the ability or potential for prosthetic ambulation that exceeds basic ambulation skills, exhibiting high impact, stress, or energy levels. Typical of the prosthetic demands of the child, active adult, or athlete.

**Table 3.** Outlined loading levels with weight limits according to the ISO (International Organization for Standardization) standard 10328: Structural testing of lower limb prostheses.

Loading level	Weight limit (up to, kg)
P3	60
P4	80
P5	100
P6	125
P7	150
P8	175

### Mechanical Performance Requirements of Load-Bearing Lower Limb Prostheses

The scope of ISO 10328 states that the standard is “suitable for the assessment of the conformity of lower limb prosthetic devices or structures, with the strength requirements specified in 4.4 of ISO 22523:2006” [36].

The alignment of loading in this standard can be set up in 2 separate test conditions (conditions 1 and 2). These alignments represent the heel strike and toe-off gait phases of walking, respectively. The alignments remain constant for all variations of loading levels. Early studies show Current et al [37] adhering to the testing procedures of ISO 10328 for transtibial prosthetic sockets in the 90s.

**Figure 4.**
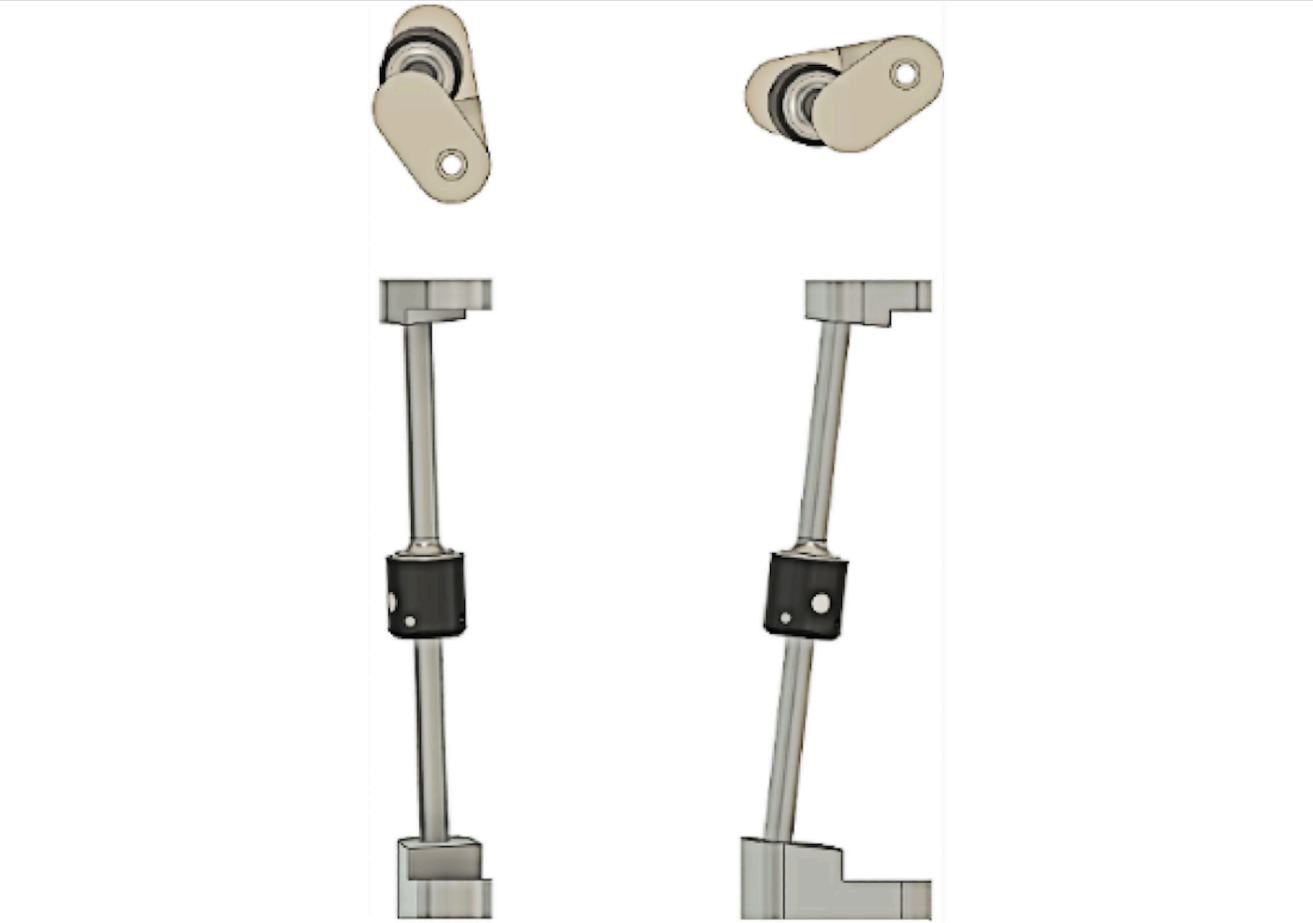
An example of a testing configuration for condition 2 of the test standard ISO (International Organization for Standardization) 10328: structural testing of lower limb prostheses (digital simulation render).

Alignment offsets to determine the condition’s loading line are marked at the heights of specific anatomical landmarks, such as the ankle and knee center. These alignment requirements, however, do not account for the complex alignment requirements of each individual patient limb and socket. A visual of the alignment requirements for a custom test rig for condition 2 of ISO 10328 can be seen in Figure 4.

A breakdown of ISO 10328’s structural tests includes the following [36]:

1. Proof test: The ability for a device to withstand a load capacity relevant to its determined loading level and device complexity, with no deformation above 5 mm for a total predetermined device length.
2. Ultimate strength test: The device is tested once until failure or until it has reached the maximum load capacity as determined by the standard.
3. Fatigue test: The device is tested cyclically using an outlined pulsating test force for a prescribed number of cycles, determined by the loading level and device complexity used.

The standard also outlines requirements for torsional testing, which is a common cause of lower limb prosthesis failure as investigated by Heitzmann et al [38] and Quinlan et al [39] in separate studies. Another common point of failure due to loading is observed at the socket’s distal end. Failure here is typically due to the concentration of forces at connection points made between the custom-made socket and CE-marked componentry of adapters (leading to the pylon and foot of the device [40]. Throughout ISO 10328, there is no consideration for the prosthetic socket component wall thickness, alignment, or structural design complexity, regardless of their impact on mechanical and structural efficacy.

Other test requirements outlined in ISO 22523 include the following for lower limb prostheses [32]: (1) flammability, toxicity, and biocompatibility of materials; (2) infection and microbiological contamination; (3) corrosion and degradation resistance; and (4) protocol on design and mechanical requirements of the device.

ISO 22523 also states that safety factors corresponding to the particular use of a lower limb prosthesis are determined “by the ratio between the test loading conditions and/or test loading levels applied to the device and the corresponding loads expected to be exerted on the device during use” [32].

Device failure has a direct and immediate result on the quality of the life of the wearer, be it structural or chemical. If a device fails, it takes time and clinical capacity to replace the device, meaning a patient may not have an immediate solution. By performing these tests and forecasting the applications of new technologies in the industry, devices are provided to wearers in the most efficient and safe manner, with security and sustainability in mind.

While there are challenges surrounding the efficacy of test standards for transtibial sockets, the socket itself has evolved and continues to increase in strength, material, and form with technological advancements. The aim of this review is to highlight advances and advantages of innovative technology adaptations to the field by understanding both traditional and digitalized methods. The focus of the review is specifically for the manufacture of load-bearing transtibial prosthetic sockets. This review pertains to an equal focus on clinical applicability, regulatory challenges, and mechanical performance of these sockets.

## Methods

This state-of-the-art review was conducted by using appropriate keywords across a multitude of reputable and peer-reviewed databases to identify relevant publications made over the last 25 years. In accordance with the Introduction section of this review, the focus of these keywords was equally divided between the topics of clinical applicability, regulatory challenges, and mechanical performance of load-bearing lower limb sockets. The key subheadings that were identified for the purposes of the communication of results were as follows:

1. Current relevant materials.
2. Current AM technologies for load-bearing socket production.
3. Current structural design and testing approaches for load-bearing sockets.
4. Clinician and technician experience in the 3D printing of load-bearing prosthetic sockets.

A collection of publications was compiled and separated according to their relevance to each of the aforementioned subheadings of the “Results” section. Knowledge and literature gaps were identified and stated in the “Discussion” section of this review upon analysis, with recommendations for future studies and consideration also offered.

Technical information and prices were referenced for specific AM solutions from relevant companies engaged in the AM of prosthetic load-bearing sockets from the years 2022 and 2023. This was carried out to provide further context to the current state of technology in an economic environment. These insights were compiled according to their technological solution and compared for their strengths, weaknesses, and clinical relevance for load-bearing prostheses.

## Results

### Current Relevant Materials for Load-Bearing Socket Production

Comparable material properties of traditionally laminated sockets are difficult to produce due to the nature of the socket itself. The customized design approach for each individual patient, combined with the nonstandardized use of specific ratios and material combinations, results in the lack of identifiable durability readings for a prosthesis. Activity levels and weight limits further complicate matters. For example, a socket without carbon fiber may support one person; however, if activity level increases, this socket may be considered redundant. As activity levels increase, there may be further requirements, such as material additions, overall mass, and thickness of the socket itself.

Current AM material solutions most commonly explored for load-bearing sockets consist of polypropylene, polylactic acid (PLA), polyamide 12, and carbon fiber reinforced composites [12,41]. The method of AM used to manufacture devices from these materials also has an impact on the structural and chemical integrity of the final device. As AM typically relies on the adhesion of layers of material to build a 3D form, there is an added risk of delamination dependent on preloaded process parameters. Print orientation will also add risk of delamination dependent on the loading applied to the printed socket. The problem of delamination is most commonly seen to arise when using fused filament fabrication (FFF) solutions, which are defined and discussed in the following section [42]. Other solutions, such as selective laser sintering (SLS) and Multi Jet Fusion (MJF), result in more efficient layer adhesion compared to FFF (also discussed in the following section) [43].

### Current AM Technologies for Load-Bearing Socket Production

#### Overview

For the AM of prosthetic sockets, research has concentrated on polymer-based approaches due to cost, weight, and similarity of resins or postprocessing to existing applications. The following four forms of AM will be the focus of this review: (1) FFF, (2) stereolithography, (3) powder bed fusion, and (4) binder jetting.

Table 4 highlights the comparative insights of each, with the following relevant to further details and technical processes of each.

**Table 4.** Comparative insights of fused filament fabrication, stereolithography, powder bed fusion (selective laser sintering), and binder jetting (Multi Jet Fusion) for prosthetic lower limb socket production.

Solution	Strengths	Weaknesses	Clinical relevance
FFF^a^	Highly accessible, cheaper option.	Inferior mechanical properties for more accessible material options.	Entry point to 3D printing, highly relevant for prototyping and concept ideation.
SLA^b^	High-resolution prints offer more detail in prints.	Postprocessing requirements are essential for biocompatibility and mechanical properties.	Most accurate printing solution but higher risk of technical and patient contact with harmful substances.
SLS^c^	Strong parts are more suitable for load-bearing applications.	Expensive, large machinery, and time-consuming.	More accessible option for load-bearing 3D printing than MJF^d^.
MJF	Higher isotropic properties than SLS.	Limited material and color choices for finished print.	Reliable for load-bearing applications.

a FFF: fused filament fabrication.

b SLA: stereolithography.

c SLS: selective laser sintering.

d MJF: Multi Jet Fusion.

#### Fused Filament Fabrication

FFF is the most common method of commercial 3D printing and is one of the cheapest methods of AM. This process involves the melting of material extruded through a heated nozzle and layering these extrusions to form a 3D shape [44].

FFF printing has been used to create load-bearing transtibial sockets using materials, such as polypropylene, PLA, and carbon fiber–reinforced PLA [12]. This technology also has the ability to create accessory components to load-bearing prostheses, such as socket liners from thermoplastic polyurethane material [45].

Where FFF-printed sockets have been tested to compare with traditionally made load-bearing sockets, results concluded that an FFF-printed socket was at higher failure risk than traditionally made sockets at higher loading weights and activity levels [12]. The process involved with FFF production also leaves vulnerabilities in layer line adhesion and resolution in comparison to other processes. FFF layers can appear as visible weld lines, prone to delamination dependent on the stresses that are applied to the print [46]. As a high percentage of prosthesis wearers are those who experience symptoms of diabetes and are prone to infection, it is best practice to minimize bacteria growth on a prosthesis. Performing postprocessing can decrease the potential buildup of bacteria that may accumulate between the crevices of layer lines [47]. As a result, postprocessing practices are often implemented to obtain a more homogeneous surface on FFF-manufactured sockets.

#### Stereolithography

Kim et al [48] showed that stereolithography printers use lasers to cure resin one layer at a time to create a 3D structure, making it one of the most accurate printers on the market for socket production. If produced correctly, a stereolithography-manufactured device can display promising mechanical properties for load-bearing socket purposes that can often supersede FFF applications. However, the manufacturing and postprocessing procedure can result in a more brittle final component if not produced correctly [49]. Although there are biocompatible material solutions available for prosthetic socket production, improper stereolithography curing and postprocessing can still result in harmful material contact with human skin. This hazard has the potential to impact not only the operator of the technology but also the wearer of the prosthesis. As such, biocompatibility concerns are often brought to the forefront for stereolithography production [50]. Stereolithography printing is recognized in this paper as a commonly known and accessible AM process; however, the literature surrounding its use to produce load-bearing lower limb sockets is limited when compared to FFF, SLS, and MJF solutions in research. Improper postprocessing and curing also have a greater effect on device properties for stereolithography than that of other AM solutions. However, it is important to note that disparity or misalignment with processing requirements in any AM or traditional process can have a detrimental effect on the efficacy of the manufactured device without isolating the stereolithography solution.

#### Powder Bed Fusion

**Figure 5.**
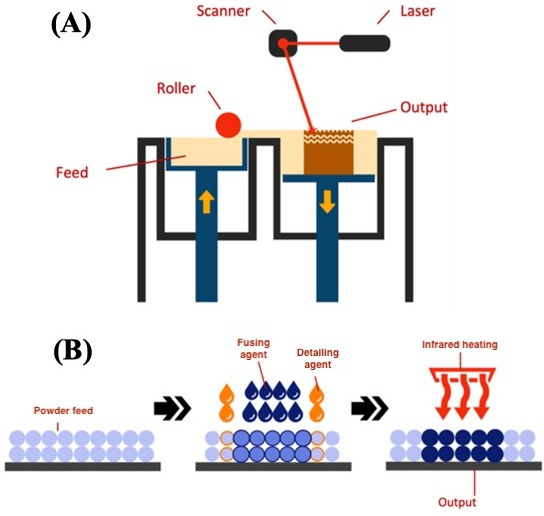
Breakdown of selective laser sintering: (**A**) Multi Jet Fusion and (**B**) polymer printing solutions.

Powder bed fusion by laying and curing thin layers of powder material to create a 3D form. One method of using this powder approach lies in SLS, where Budinski et al [46] describe how a UV beam cures each layer in a predetermined path to create the 3D form (refer to Figure 5A) [51]. Postprocessing requirements for SLS involve cleaning the print from excess powder, tumbling the print for a smooth finish, and possibly applying finishing dyes or coatings.

The strength of SLS-printed parts exceeds those of FFF and stereolithography alternatives, making it an AM solution worthy of definitive use for a variety of applications [52]. These applications can be seen throughout the medical, automotive, and aerospace industries [53]. With the ability to recycle unused powder and the freedom of printing without supports accessed through this solution, SLS printing is an attractive solution when mechanical strength and sustainability are necessary for their application [54]. The disadvantages with SLS printing, however, come from the limitations of material choice, costs, and postprocessing [52,55]. Both material and color choices are limited, yet are currently being explored through composites that need extra attention in their application [56]. Postprocessing of parts can result in hours of work due to parts cooling and manual cleaning requirements [57].

SLS-printed sockets have been deemed to be an identifiable solution for the manufacture of patellar-bearing socket solutions for transtibial amputees due to their high strength [58]. Sockets for load-bearing solutions have been created and are commercially viable. However, initial research into the device design shows that there is work to be done on the distal end of the socket [59]. Time frame and conditions required for SLS printing may also be a challenge for P&O companies, as there are long and regulated cooldown requirements before the part can be postprocessed to avoid issues such as part warpage.

#### Multi Jet Fusion

Guo et al [52] outline binder jetting as similar to powder bed fusion; however, the final curing process involves the addition of a liquid binding agent to fuse each layer together [60]. Figure 5 (B) visually outlines the differences between the 2 solutions for further context, with MJF representing binder jetting technology through.

MJF’s nylon materials are almost identical to those of SLS nylon, with the advantage of being up to 25% more ductile in the Z axis of prints [60]. MJF printing solutions are currently being used for load-bearing prostheses and have been tested to show similar strength to that of traditionally made sockets [10]. The testing used to show this, however, is varied across the literature. In the case of Ráž et al [10], the sockets tested were laid horizontally, and an axial force was applied to an exterior wall. This is not reflective of everyday use, where a socket would be in an approximate vertical position as a user is walking, running, or jumping.

### Current Structural Design and Testing Approaches for Load-Bearing Sockets

There have been several studies over the past few decades on AM of prosthetic sockets. However, fewer are available that report the success of load-bearing prostheses. Many initial studies focus on the software design optimization to improve the finished socket component by increasing wall thicknesses and adding reinforcements to the 3D-printed socket exterior [28,61,62]. Others, such as Hsu et al [63] would later go on to improve the strength of printed sockets by using resin coatings [52,63].

Kim et al [12] could conclude from data collected in their systematic review that “failure mainly occurred at the distal end of the socket or the pyramid attachment […] whether a laminated composite socket or a 3D printed socket”. This is consistent with the ISO 10328 testing protocol. Although design modifications can improve socket strength in digitalized workflows, there are no clear limitations on maximum socket density, wall thickness, or viable mounting or assembly components and solutions for the socket.

When reviewing a variety of prosthetic socket testing papers and abstracts, it is apparent that the most logical approach to structural testing is to use the standard ISO 10328. However, there is a disparity in test setup approaches across the literature. This is apparent in the componentry used not only in the assembly of the prosthesis, but also in the attachment of the prosthesis to the test machine. The American Prosthetic and Orthotic Association recently published findings of key areas that are in need of attention when considering the creation of a standardized testing solution for prosthetic sockets [62]. The findings of this paper outline the need to represent patients who lie outside the “norm” for prosthetic socket shape and loading. They also highlight the need to determine appropriate loading conditions that will suitably reflect all socket types. Taking these findings into consideration, it would be suggested that more than one test setup would be required in order to capture a broader range of patient alignment, physical form, and loading parameters.

Assuming the requirements in ISO standards 10,328 and 22,523 are met during testing, the structural and physical makeup of a 3D-printed load-bearing transtibial prosthetic socket is valid and acceptable for use. However, including a socket in testing according to ISO 10328 presents complications in designing a test rig that ensures consistent alignment. Many studies have tested transtibial sockets with this standard, although each test rig used is custom-made and varies between studies [10-12,37,39-42,48]. As per the nature of socket loading in a real scenario, forces applied to the internal socket surface are transferred through the soft tissue of the residual limb. It is difficult to translate these loading conditions into a repeatable test rig. As such, rigid or foam structures are typically used in custom test rigs as a replacement for the residual limb during testing. This draws even more disparity between not only each study completed but also between testing and real-life scenarios [11,40]. Concerns are raised for the reliability of comparing or correlating results through systematic review, as each custom test rig uses different attachment components and applies different forces to the internal socket [39,48].

Numerous papers suggest the replacement of the prosthetic foot with custom-made fixtures made of metals from aluminum to steel, as a prosthetic foot alters the moments acting on the specimen. In the ISO standard 10328, the load line (or alignment offsets) for condition 1 (heel strike) passes through the distal end of the socket, whereas the load line for condition 2 (toe-off) passes anterior to the socket distal end. Condition 2 generates larger moments at the distal end of the sockets, representing the worst-case scenario load [36]. To lessen moments caused by fixed components, Owen and DesJardins [61] use ball joints in the upper and lower loading fixtures during ISO 10328 testing [42]. It is also seen in this paper that, although both conditions 1 and 2 were tested for proof strength, only ultimate strength tests performed with condition 2 were communicated to readers.

The use of finite element analysis (FEA) is prevalent in the studies involving the production and testing of load-bearing prosthetic sockets [10,54,64]. Using FEA modeling for prosthetic testing can reduce the requirement of clinical appointments for an end user, further promoting a hygienic and efficient workflow of manufacturing, fitting, and design [54]. Through FEA evaluation, detailed information on internal and external tissue loading (which can be responsible for discomfort and injury) is made available [65]. By incorporating FEA into the structural design and form of the socket, researchers have reduced the potential weight and material use required for creating a lower limb prosthesis, focusing on a monocoque design [66].

SLS-printed sockets have been observed to fail when socket design in the digitalization space is altered to improve efficiency. These alterations sacrifice material or design quality to optimize material usage. As such, it has been noted that parts produced with SLS technology must be produced at the highest density outcome to minimize failure [67].

Software solutions for the structural design of the prosthetic socket are constantly being explored, as there is a significant bridge to gap between engineering design approaches and clinical expertise. It is difficult to incorporate patient comfort, pressure relief, and the understanding of patient alignment when engineering a digital file [68]. Optimal design solutions would see a simple, step-by-step process wherein artificial intelligence would accommodate for patient landmarks and clinical expertise to automatically create an ideal socket form for a patient.

An important result from literature on structural testing for load-bearing sockets is that failure mode is commonly identified at the distal end of a socket for both traditionally made and 3D-printed sockets. In the cases of 3D-printed sockets, delamination of print layers is also identified as a primary failure mode [44,69]. Kermavnar et al [70] have noted that SLS-printed prosthetic sockets may also leave a grainy surface finish, which may be undesirable for sensitive skin contact or regular movement of the body. However, it is possible that this issue can be rectified with postprocessing the printed socket.

### Clinician and Technician Experience in the 3D Printing of Load-Bearing Prosthetic Sockets

The practices of prosthetists and orthotists are accredited through 14 education standards outlined by the International Association of Prosthetists and Orthotists (ISPO) or through the university courses themselves [15]. Where a clinician is required to achieve a minimum level of 6 on the European Qualification Framework, a technician (who works outside the presence of the patient in the manufacturing process) only requires a minimum level of 4. Despite technological advancements, the current clinical and technical education system does not incorporate AM knowledge or experience into its learning outcomes. Educational systems have also not adopted 3D scanning or digital rectification processes as outputs to module learnings. Any experience of 3D printing, its materials, or the digital manufacturing process for P&O must be obtained through external training services provided by digital manufacturers. Other than this, typical clinician and technician experience with 3D printing will stem from hobbies outside of working hours. For the future of prosthetic education, Spaulding et al [71] call for “a more evidence-based approach to applying new technology and materials” in their considerations.

Although there has been an increase in the adoption of AM in the prosthetic industry, there are limited controls implemented for product quality. The World Health Organization has incorporated 3D printing into their regulations of lower limb prostheses and recommends the adherence to quality standard ISO 13485. However, little guidance is given for the validation and monitoring of AM sockets [33,72]. Despite these challenges, there have been successful rates of adoption for digitalization internationally. Low-income countries have seen benefits from the accessibility of 3D printing and scanning through voluntary organizations [73]. In a direct clinical setting, business process reengineering has been analyzed for the digitalization of the prosthetic socket industry by Mohamed et al [74]. It was found that “implementing digitalization in the fabrication process can significantly address the bottlenecks identified in the current business model” for prosthetic socket production. Another paper by Gutierrez [75] highlights the reduction in manufacturing time when switching to digitalized workflows and providing “clinicians with additional time to dedicate to patient care and improved outcomes.”

## Discussion

### Principal Findings

Upon analyzing the findings in the current literature for load-bearing sockets manufactured using digitalization, the following discussion is outlined: (1) outlook on developing technologies for load-bearing prostheses; (2) recommended solutions for prosthetic socket additive manufacture; (3) recommendations for improving adoption rates of digitalized processes; (4) bias and limitations of the current literature; and (5) conclusions.

### Outlook on Developing Technologies for Load-Bearing Prostheses

The clinical and manufacturing procedures for P&O have remained virtually the same for a number of decades, with excessive cost, waste, and physical data volumes produced [76]. The materials involved with production are reliable, consistent, and measurable through approved material standards and product requirements. This reliability increases difficulties in the deviation of current to digitalized processing methods. As a result, the manufacturing process had not developed from plaster molding, resin casting, and thermoforming up until the possibilities of AM were explored [76].

All physical data imperative to produce a traditionally manufactured prosthetic socket (such as the plaster molds of the patient) must be retained for a determined period [68]. This results in the need for large storage areas and physical records. The entire fitting and manufacturing process itself can take several days to weeks, including steps from fitting the socket, assembling componentry (such as the foot and other components), and refitting, to ensure correct alignment and comfort [68]. For initial patient plaster casting, the traditional process of obtaining data for socket rectification is a time-consuming and physically invasive process.

The introduction of innovative technology has the ability to address these discussed pain points and impact the design methodology of prostheses for the better [24]. Changing to 3D scanning for form capture, digitalizing rectifications, and 3D printing final sockets are just a few examples of beneficial innovative technology implementation for sockets [68]. These evolutions have the potential to reduce financial, time, and employee resource costs if effectively executed [26]. The integration of technologies from AM, 3D scanning, and computer-aided modeling will form innovative design and production workflows. This may bring an opportunity to create new products that can be more functionally pleasing and sustainable through human-centered and emotional design practices [70]. Practices can become less physically invasive for patients, particularly through 3D scanning, resulting in greater cases of positive experiences within a clinical setting.

### Recommended Solutions for Prosthetic Socket Additive Manufacture

The manufacturing hardware mentioned in this review favors powder bed fusion and binder jetting in achieving the mechanical properties when prioritizing quality and high strength for weight-bearing devices. Out of both processes, Cai et al [60] highlight how MJF has higher ductility in the Z axis by up to 25% for nylon printed parts. They also highlight how surface finish and print accuracy were superior to SLS nylon printed parts.

With the above stated, it is important to note that although the cost of printers for SLS is more expensive than MJF, printers for MJF need a higher amount of maintenance. Parts replacement and material handling are highly demanding compared to those of SLS due to their additional use of fusing agents. The recommendations from this review are that SLS or MJF technologies accessible within a P&O company would promote adoption for new businesses investing in digitalized workflows, as their strength is comparable to traditional sockets. The possibilities of color printing are also becoming more accessible through MJF printing, and dyeing parts is possible through SLS technology.

For cheaper in-house prototyping, FFF is recommended over stereolithography due to the lack of postprocessing requirements and reduced health and safety hazards that come with stereolithography’s liquid resin curing. By prototyping in FFF before committing to SLS or MJF costs, it is possible to achieve clarity in the final component form ahead of investing greater resources and expense. By using a prototyping FFF printer for prosthetic printing, it may also build user confidence and engagement with AM technologies for technicians and clinicians.

### Recommendations for Improving Adoption Rates of Digitalized Processes

There is an increased demand for security in digitalized socket manufacturing processes through mechanical testing. To achieve reliable data on this subject, the importance of replication and repetition of a singular testing method for both traditional and 3D-printed prosthetic sockets should be realized. If a standardized method for socket testing was discovered, it may have the opportunity to increase the application of both functional and emotional design opportunities through 3D printing. These emotionally driven alterations may then be tested for impact on overall device strength. This would allow for the evaluation and inclusion of custom accessory components if deemed safe after passing relevant test standards. These additions would aid the cultural adoption of device wearers, improving the device sustainability at a decreased cost in comparison to traditional cosmetic inclusions.

In mechanical testing of orthopedic implants for the hips, components are often tested to last within set condition parameters from worst-case scenario to best-case scenario. A testing procedure similar to this approach may be most useful, with sockets tested from a worst-case to best-case scenarios in terms of alignment, form, and loading parameter. If the material or process used for socket creation passes both these setups, then it may be considered that all scenarios varying between these extremes can be considered safe for use.

It is recommended that the digitalization workflow should be treated as achievable and relevant to (but not altogether a substitute for) the traditional socket workflow. Although digitalized processes have the potential to streamline socket production, they cannot replace the traditional workflow entirely for more complex patient cases. Further study is required to prove the efficacy of digitalized workflows for various cases of extreme alignment, component inclusions, and individual patient considerations.

Finally, it is important to recognize that implementing a digitalized workflow, which works in a predominantly online space, is a significant change to the know-how, skill sets, and culture, learned and lived by current clinical and technical employees. This transition should be recognized as a challenge for clinicians and technicians. It is recommended that clinical language, taught landmarks, clinical experience, and employee work ethic should be incorporated into the digitalized workflow in order to minimize abandonment of this way of working.

New employee recruits may be better suited to the adoption of digitalization due to a lack of experience working traditionally, compared to established and experienced employees. However, by building on current innovation, engaging with educational exercises on digitalization, and increasing AM accessibility, the digitalized workflow can be adopted by all within the P&O industry regardless of experience. The transition from traditional workflows to digitalized ones takes time and must be provided with appropriate support and resources by company management and the industry.

### Bias and Limitations of the Current Literature

As AM is a rapidly evolving field with gray literature and commercial data that may not be peer-reviewed, it is important to note a potential effect of bias in reviewed data sources. This review has taken care in referencing peer-reviewed literature through the exploration of case studies, AM implementation, and previous research. Some references are commercially biased for the purposes of exploring and expanding upon the current state of the art of the AM and prosthetic industry within this review.

### Conclusions

When it comes to the manufacture of prosthetic and orthotic devices, both a standardized and customized way of working converge to return a final product. If AM is to aid in the customized manufacture of components, such as prosthetic sockets, the finished component must have high tensile strength and be relatively lightweight, with ductility for potential failure and some flexibility for everyday wear. The device must also be sufficiently resistant to wear over time and have the ability to withstand varied temperature, humidity, and UV exposure. These requests are not easy to accomplish in all outlined processes.

Upon literature review for essential requirements of prosthetic lower limb devices, it has been identified that there is a distinct lack of regulation of custom-made devices in comparison to the standardized componentry used alongside them. When reviewing the possibilities of testing these custom-made components, such as the prosthetic socket, there are many challenges to face in order to achieve a true representation of the broad range of prosthetic device wearers. While research is currently being conducted using test methods on prosthetic sockets that are designed for standardized componentry, there is a need to apply regulations of socket testing to reduce disparity in testing setups, parameters, and componentry inclusion. The choice of AM solution as well as material can also significantly impact the structural integrity of the socket, as processing times, volumes, cost, strength, and layer adhesion are among the top values to be considered for each method of printing.

It is suggested that an understanding of the culture of P&O companies be captured to evaluate the true potential of AM in the prosthetic space. It is also imperative to note that although AM technologies have the scope to greatly enhance and improve the manufacturing processes of load-bearing sockets, the technical skill set and decision-making of a clinician and technician cannot be replaced and left behind, both due to their clinical knowledge or expertise and the need for further work on the efficacy of AM for load-bearing prosthetic devices.

## Abbreviations

AM: additive manufacturing
CE: Conformité Européenne
FEA: finite element analysis
FFF: fused filament fabrication
ISO: International Organization for Standardization
ISPO: International Association of Prosthetists and Orthotists
MJF: Multi Jet Fusion
P&O: prosthetics and orthotics
PLA: polylactic acid
PP: polypropylene
SLS: selective laser sintering
